# There’s a SNARC in the Size Congruity Task

**DOI:** 10.3389/fpsyg.2018.01978

**Published:** 2018-10-17

**Authors:** Tina Weis, Steffen Theobald, Andreas Schmitt, Cees van Leeuwen, Thomas Lachmann

**Affiliations:** ^1^Cognitive and Developmental Psychology, Center for Cognitive Science, University of Kaiserslautern, Kaiserslautern, Germany; ^2^Experimental Psychology Unit, KU Leuven, Leuven, Belgium

**Keywords:** number symbol, mental number line, response dimension, compatibility, ideomotor theory

## Abstract

The *size congruity* effect involves interference between numerical magnitude and physical size of visually presented numbers: *congruent* numbers (either both small or both large in numerical magnitude and physical size) are responded to faster than *incongruent* ones (small numerical magnitude/large physical size or vice versa). Besides, numerical magnitude is associated with lateralized response codes, leading to the Spatial Numerical Association of Response Codes (SNARC) effect: small numerical magnitudes are preferably responded to on the left side and large ones on the right side. Whereas size congruity effects are ascribed to interference between stimulus dimensions in the decision stage, SNARC effects are understood as (in)compatibilities in stimulus-response combinations. Accordingly, size congruity and SNARC effects were previously found to be independent in parity and in physical size judgment tasks. We investigated their dependency in numerical magnitude judgment tasks. We obtained independent size congruity and SNARC effects in these tasks and replicated this observation for the parity judgment task. The results confirm and extend the notion that size congruity and SNARC effects operate in different representational spaces. We discuss possible implications for number representation.

## Introduction

Which is larger: the number of stars in the galaxy or the number printed on LA Galaxy star’s team shirts? This question might lead to some confusion because of the conflated ways in which the numbers could be considered large or small, i.e., printed size and numerical magnitude. This kind of incidental conflation has a counterpart in the well-known *size* *congruity effect* in speeded choice reaction times (RTs) experiments (Paivio, 1975; Banks and Flora, 1977; Besner and Coltheart, 1979; Henik and Tzelgov, 1982;; Schwarz and Heinze, 1998; Schwarz and Ischebeck, 2003). Size congruity experiments invariably show that it is easier to respond to a small numerical magnitude printed in small physical size or a large numerical magnitude printed in large physical size, i.e., a *congruent* stimulus, as compared to an *incongruent* one (a small numerical magnitude printed in large physical size or vice versa).

Size congruity effects occur both when single numbers are judged against a given standard (Schwarz and Heinze, 1998; Schwarz and Ischebeck, 2003) and in paired comparison tasks (Besner and Coltheart, 1979; Henik and Tzelgov, 1982). Moreover, they occur, regardless of whether the target dimension is physical size (Henik and Tzelgov, 1982) or numerical magnitude (Besner and Coltheart, 1979) or neither, e.g., when participants decide about the parity of the numbers (Viarouge et al., 2014). This result suggests that both stimulus dimensions are activated by the stimuli irrespective of their relevance, leading to interference akin to the Stroop effect (Stroop, 1935).

Contrasting hypotheses on the locus of interference were offered by Schwarz and Heinze (1998): the *shared representation* account and the *shared decision* account. According to the first, the dimensions of numerical magnitude and physical size share a common representation, which enables cross-talk between these dimensions during stimulus encoding. According to the second, numerical magnitude and physical size are processed in parallel, i.e., in functionally independent channels. Both can activate different response tendencies that interact at the decision level. Santens and Verguts (2011) modeled both hypotheses on their data and concluded in favor of the shared decision account. This conclusion is consistent with ERP data by Schwarz and Heinze (1998), who reported an effect of congruity on the N2 evoked potential in frontal and mid-central electrodes, as well as with fMRI results by Kaufmann et al. (2005) and Ansari et al. (2006) who observed higher neural activity for incongruent trials in prefrontal cortex and anterior cingulate cortex. Even though the conflict arises in a relatively late stage of processing, it remains a conflict arising spontaneously between stimulus features. These are processed in parallel up to and including the decision making process, where they give rise to conflicting response tendencies.

Such type of interference between stimulus dimensions may be distinguished from effects relating to compatibility of stimulus and response. Generally, choice responses are faster and more accurate when stimulus and response share a similar feature, e.g., placement to the same side of the participant, i.e., both on the left or both on the right – compatible –, as opposed to one on the right and the other on the left – incompatible. In choice reaction time experiments, if the one stimulus code is predominantly associated with the left response and the other with the right response, lateralized responses on the associated, compatible side are favored (Fitts and Seeger, 1953).

There are reasons to assume that number stimuli are associated with lateralized responses. Dehaene et al. (1993) showed that smaller numbers (e.g., 1, 2) were responded to faster with the left hand, whereas larger numbers (e.g., 8, 9) were responded to faster with the right hand. This Spatial Numerical Association of Response Codes (SNARC) effect occurred even in a parity judgment task. SNARC-like effects are rather wide-spread and not limited to numerical magnitudes. Spatial association effects exist also for letters of the alphabet (Gevers et al., 2003), months of the year and days of the week (Gevers et al., 2004), pitch of a stimulus (SPARC; e.g., Weis et al., 2016), loudness (Hartmann and Mast, 2017), luminance (Fumarola et al., 2014), temporal duration (Vallesi et al., 2008; Prpic et al., 2016), angle size (Fumarola et al., 2016), and were even shown for conceptual magnitude (animal names; Shaki et al., 2012).

SNARC and size congruity effects can be observed jointly in choice response experiments (Fitousi et al., 2009). To observe size congruity, in these experiments left and right hand responses are typically pooled, thereby balancing SNARC compatible and incompatible conditions. In Experiment 1 of Fitousi et al. (2009), the authors showed that, when responding hand (response side) was taken as an independent factor in physical size and parity judgment tasks, both size congruity and SNARC effects occurred independently, i.e., without interaction. They took their result as supporting the notion that size congruity and SNARC effects belong to distinct components of visual information processing. In particular, the results suggest that size congruity effects are associated with the “what” system, while the SNARC effect is associated with the “where” system, as the latter is relevant to motor action (see Goodale and Milner, 1992, for a review on separate visual systems).

Fitousi et al. (2009) considered the relationship of size congruity and SNARC effects in physical size and parity judgment tasks, but not in the numerical magnitude judgment task. Our aim is to replicate their parity judgment task and extend their study with a magnitude judgment task. If Fitousi et al. (2009) are correct about the independency of both effects, we may expect independence also for the numerical magnitude task. We might, however, expect a different outcome with numerical magnitude as the focus of the task. This is because, as the SNARC effect shows, numerical magnitude is relevant to the action system.

We know of only one case where a SNARC-like effect has been found involving physical size. Ren et al. (2011) observed that participants responded faster with the left hand if a small disk was presented and faster with the right hand if a large disk was presented. We may call this special case the Spatial Size Association of Response Codes (SSARC) effect.

The SSARC effect in Ren et al. (2011) may be action-related; for instance, grasping the larger (heavy) disk with the preferred (right) hand. To our knowledge, a SSARC effect has not been observed for numbers. Physical size, therefore, may be neutral to the action system, unlike numerical magnitude. Thus, dependencies between SNARC and congruity might well arise, once numerical magnitude instead of physical size is the focus of the task.

The action-relevance of numerical magnitude is likely to affect the stimulus representation, according to current ideomotor theory (Hommel et al., 2001). Its key assumption is that perception shares a common code with motor behavior: motor actions are represented by their perceivable effects (Shin et al., 2010). Whereas stage-wise processing models (Sternberg, 2018) will expect motor effects exclusively in late processing stages, e.g., response preparation or execution (Pashler, 1984; Ruthruff et al., 1995), ideomotor theory explains them from early stages on, based on features shared between stimulus and action effect codes.

The Theory of Magnitude (ATOM, Walsh, 2003) assumes a unifying concept of Spatial Quantity Association of Response Codes (SQUARC). Although nominally a “joint representations” account, the conflict is assumed to occur at later stages of processing (e.g., Schwarz and Heinze, 1998) and is therefore independent of the input format as long as a metric of time, space or quantity can be attributed to the stimulus.

**FIGURE 1:**
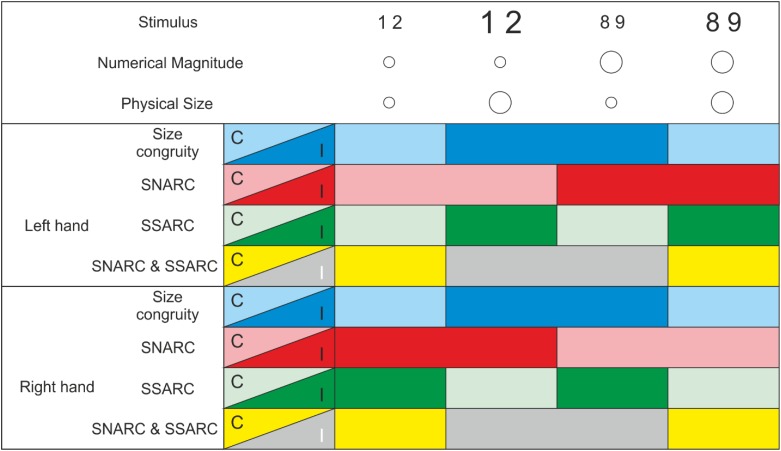
For the different stimulus conditions [numerical magnitude: small (1,2) and large (8,9); physical size: small and large] the respective compatible (C, light colors) and incompatible (I, dark colors) conditions for each responding side (left and right hand) were depicted. Size congruity only depends on numerical magnitude and physical size and is independent of the responding hand; i.e. if both stimulus features (physical size and numerical magnitude) are small, or both of them are large, this corresponds to the congruent condition (light blue), whereas if both have different sizes, this corresponds to the incongruent condition (dark blue). The SNARC effect depends on the responding hand and numerical magnitude, independent of the physical size. The SNARC is compatible (light red) if small numbers (1 and 2) have to be responded with the left hand and large numbers (8 and 9) have to be responded with the right hand. Consequently, the SNARC is incompatible (dark red) if small numbers have to be responded with the right hand and large numbers with the left hand. Similarly, the SSARC effect which depends on the responding hand and the physical size of the stimuli, independent of the numerical magnitude. The SSARC is compatible (light green) if numbers in a physically small numbers have to be responded with the left hand and physically large numbers have to be responded with the right hand. Consequently, the SSARC is incompatible (dark green) if physically small numbers have to be responded with the right hand and physically large numbers with the left hand. The combination of both, the SNARC and the SSARC results in the size congruity effect (SNARC and SSARC, yellow and gray), i.e., if both effects are compatible or both are incompatible, they are congruent (yellow or light blue), if one is compatible and the other is incompatible they are incongruent (gray or dark blue).

*Anticipated* effect codes can influence perceptual and cognitive coding (Nikolaev et al., 2008; Harrison and Ziessler, 2016). SNARC incompatibility may lead to anticipation of a conflicted response; this anticipated conflict may enter into the representation and the decision making, where it can moderate the incongruity effect. As a result, an interaction of SNARC and size congruity effects would appear. Our aim, therefore, is to test whether SNARC effects in the numerical magnitude task interact with congruity effects. As in a regular size congruity experiment, large and small numerical magnitudes are presented in large or small physical sizes (i.e., fonts). The task is to decide (button press) whether a stimulus is larger or smaller in numerical magnitude than a given standard. This, however, is done in separate runs in SNARC compatible (small-left, large-right) or incompatible (small-right, large-left) response conditions. Our main interest is whether SNARC effects interact with size congruity effects. A 2 × 2 × 2 factorial design might seem appropriate, in which SNARC (in)compatibility, SSARC (in)compatibility, and size congruity are independent factors. Unfortunately, such a design is impossible, because conditions are interdependent. This can be seen most easily by starting from a balanced 2 × 2 design with two factors: SNARC (in)compatibility and SSARC (in)compatibility. We cannot add size congruity as a third factor because, by definition, half of the stimuli are already size-congruent and the other half size-incongruent: Of the size-congruent stimuli, by definition, half are both SNARC and SSARC compatible while the other half are both SNARC and SSARC incompatible; of the size-incongruent stimuli, half are ones that are SNARC compatible and SSARC incompatible and the other half are SNARC incompatible and SSARC compatible (see **Figure 1**).

We therefore chose a design including factors *Numerical Magnitude* (small vs. large), *Physical Size* (small vs. large), and *Responding Hand* (left vs. right). Consequently, cross-over interaction between Physical Size and Numerical Magnitude will amount to the size congruity effect. The SNARC effect can be understood as a cross-over interaction between Responding Hand and Numerical Magnitude, whereas the SSARC effect is represented as a cross-over interaction between Responding Hand and Physical Size. We argue that if no further interactions occur, this would accord to the notion that SNARC and SSARC effects do not interact with size congruity.

Whereas the three factors Numerical Magnitude, Physical Size, and Responding Hand are central to our study, two more factors relevant to SNARC and SSARC effects were added to the design: *Task* and *Response Dimension*. Note that because of the dependency between size congruity, SNARC, and SSARC, an effect of congruity, for instance, is indistinguishable from a cross-over interaction between SNARC and SSARC compatibility. The additional factors might help resolve such ambiguity. The influence of Task on the SNARC effect, for instance, is quite characteristic and well-known. SNARC effects are observed both in explicit (magnitude judgment) and implicit (parity judgment) task conditions (Dehaene et al., 1993), and sometimes even more strongly in the latter (Weis et al., 2016). If similar is observed here, we have supporting evidence for the attribution of our results to a SNARC effect. For this reason, besides a magnitude judgment task, also a parity judgment task was performed. Again, compatible and incompatible conditions varied between runs. In one run participants responded with odd-left/even-right and in the other run with the opposite assignment.

We also consider the possibility of a SSARC effect, i.e., an advantage for numbers printed in small physical size to the left hand and numbers in large physical size to the right hand. In contrast to the studies by Fitousi et al. (2009), Ren et al. (2011), and Shaki et al. (2012), the SSARC effect was tested only implicitly, i.e., there is no physical size judgment task. Please note that in a recent review the authors found smaller effect sizes for non-numerical SNARC-like effects when tested implicitly (Macnamara et al., 2018), which is in contrast to the SNARC effects where implicit conditions show even stronger effects (Weis et al., 2016). Nevertheless, in our design we tested the SSARC effect only in implicit conditions, in line with our previous results. To evaluate the attribution of our results to SNARC and SSARC effects, we introduced, in addition to the horizontal response dimension, a *vertical* response dimension. Vertical SNARC effects were sometimes found to be equally strong as horizontal ones (Fias and Fischer, 2005; Gevers et al., 2006a) but not always (Ito and Hatta, 2004; Holmes and Lourenco, 2012; Hartmann et al., 2014), as the mental number line may be preferentially associated with the horizontal orientation; we might expect a SSARC effect to be stronger on a vertical scale, as size is typically expressed vertically.

In sum, we predict in our experiment: a size congruity effect, SNARC and possibly SSARC effects. Interactions between size congruity and SNARC may be interpreted in terms of ideomotor theory while the absence of such an interaction would suggest that SNARC effects and size congruity effects belong to different representational spaces (Fitousi et al., 2009), even when numerical magnitude is the focus of the task.

## Materials and Methods

### Participants

Twenty-four healthy students (13 females, age range = 21–31 years, average age = 24.6 ± 2.8 years) were paid for their participation in the experiments. All participants were right-handed, had normal or corrected to normal vision and were native speakers of a language that writes letters and numbers from left to right. The protocol was approved by the ethical committee of the Department of Social Science of the University of Kaiserslautern. All participants gave written informed consent in accordance with the Declaration of Helsinki (World Medical Association, 2008). One participant (female) was excluded from further analyses due to technical problems during the measurement, and another participant (male) attended the first session only. Therefore, data of 22 participants remained for analyses. We declare that there is no conflict of interest.

### Apparatus, Material and Procedure

In two counterbalanced sessions held on separate days, participants solved a magnitude and a parity judgment task. In separate blocks of the magnitude judgment task, participants were asked in one block to respond to small numerical magnitudes (<5) with the left index finger and to large numerical magnitudes (>5) with the right index finger, and vice versa in another block. In separate blocks of the parity judgment task participants judged if a number was either even or odd; in one block, even numbers were responded to with the right index finger and odd numbers with the left index finger, and vice versa in the other block.

Both tasks, magnitude and parity judgment, were performed in separate blocks, either in a horizontal response arrangement, in which participants pressed “Q” with the left index finger and “P” with the right index finger, or in a vertical response arrangement, in which they pressed “6” with the right index finger and “B” with the left index finger on a QWERTZ keyboard. Thus, for each task, there were 2 (response assignments to left/right index finger) × 2 (vertical vs. horizontal alignment of response keys) = 4 different blocks. Each participant solved these blocks in a random order, independently drawn without replacement from the 4! = 24 possible orderings.

Each block contained 160 experimental trials, i.e., 10 repetitions of 16 different digits, which were visually presented on a computer screen in digital print. Their numerical magnitudes were identical to Weis et al. (2016), whereas their physical sizes were adopted from Santens and Verguts (2011). Four numerical magnitudes were used: 1, 2, 8, and 9, in four different physical sizes: 2.7, 5.4, 9.4, and 13.3 degrees of visual angle, resulting in the 16 different stimuli. The presence of different physical sizes was never brought to participants’ attention. The order of presentation was randomized.

In each trial a white fixation cross (1 degree visual angle) was presented for 250 ms against a black background, followed by 200 ms of black screen before stimulus presentation (white against black background) for up to 2500 ms or until response, followed by a 1000 ms inter stimulus interval.

In both tasks, small numbers (1 and 2) responded to with the left/lower key and large numbers (8 and 9) responded to with the right/upper key are regarded as SNARC compatible. The reverse mappings (1 and 2 to a right/upper key and 8 and 9 to a left/lower key) are regarded as SNARC incompatible. For the SSARC compatibility, small physical sizes responded with the left/lower key and large physical sizes responded to with the right/upper key are regarded as SSARC compatible. The reverse mappings are regarded as SSARC incompatible.

At the beginning of each block, participants were given written instructions and performed 16 practice trials (one for each stimulus type) to familiarize themselves with the task. During the practice trials, participants received feedback (correctness and reaction time) about their performance, presented on the screen for 2000 ms. Participants were told to respond as fast and accurately as possible.

All experiments were performed in a dimly lit sound proof booth using a Samsung R590 laptop. The stimuli were presented on a 15.6 inch screen with a resolution of 1366 × 768 pixels and 60 Hz refresh rate at maximum brightness at approximately 60 cm distance. The program used for stimuli presentation and response recording was Psychopy 1.8 [University of Nottingham; (Peirce, 2007)]. Each session (4 blocks × 160 trials = 640 trials in total) took approximately 45 min, including instruction and training.

### Data Analyses

There was no evidence for a speed-accuracy tradeoff, *r*(22) = −0.37, *p* = 0.08, nevertheless, RTs for correct responses and error rates (ER) are reported. A Kolmogorov-Smirnov test was used to test for normality on the dependent variable RT, *d*(22) = 0.113, *p* > 0.05, and ER, *d*(22) = 0.115, *p* > 0.05, indicating that the data did not deviate from the normal distribution.

We pooled small (1 and 2) and large numerical magnitudes (8 and 9) as well as small (2.7 and 5.4) and large physical sizes (9.4 and 13.3) and calculated a 2 × 2 × 2 × 2 × 2 ANOVA with the within-participants’ factors: *Task* (magnitude judgment vs. parity judgment), *Response Dimension* (horizontal vs. vertical), *Responding Hand* (no cross over with response side: left hand = left response side vs. right hand = right response side), *Numerical Magnitude* (small vs. large), and *Physical Size* (small vs. large) for RTs and arcsine-transformed ER. If null effect are interpreted, these were tested using Bayes factors (BF).

As shown in **Figure 1**, SNARC (light and dark red) is represented by the interaction of *Numerical Magnitude* and *Responding Hand*, whereas SSARC (light and dark green) is represented by the interaction of *Physical Size* and *Responding Hand*. In contrast, size congruity (light and dark blue) is independent of the *Responding Hand* and is represented by the interaction between *Numerical Magnitude* and *Physical Size*. Thus, the interaction of SNARC and SSARC is principally indistinguishable from the size congruity effect.

## Results

Analyses of RTs showed a main effect of *Task*, *F*(1,21) = 42.18, *p* < 0.001, $\eta_{p}^{2}$ = 0.668, with faster RTs in the magnitude judgment task (430 ms) compared to the parity judgment task (494 ms), a main effect of *Responding Hand*, *F*(1,21) = 7.30, *p* = 0.013, $\eta_{p}^{2}$ = 0.258, with faster right hand responses (459 ms), compared to left hand responses (465 ms) and a main effect of *Numerical Magnitude*, *F*(1,21) = 11.61, *p* = 0.003, $\eta_{p}^{2}$ = 0.356, with faster responses to smaller (458 ms) compared to larger numerical magnitudes (465 ms).

**FIGURE 2:**
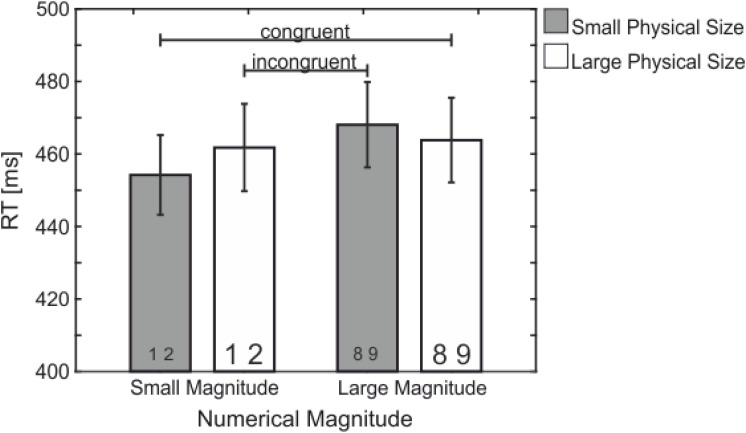
Mean RT (in ms) showing the significant interaction between Numerical Magnitude and Physical Size – the size congruity effect. Congruent conditions (large magnitude and large physical size, small magnitude and small physical size) with shorter reaction times than incongruent conditions (small magnitude and large physical size, large magnitude and small physical size).

There was an interaction between *Numerical Magnitude* and *Physical Size*, *F*(1,21) = 13.05, *p* = 0.002, $\eta_{p}^{2}$ = 0.383 – the size congruity effect (see **Figures 1**, **2**) – indicating slower RT for incongruent (small numerical magnitude – large physical size: 461 ms and large numerical magnitude – small physical size: 468 ms; dark blue in **Figure 1**) as compared to congruent (small numerical magnitude – small physical size: 454 ms, large numerical magnitude – large physical size: 463 ms; light blue in **Figure 1**) trials.

**FIGURE 3:**
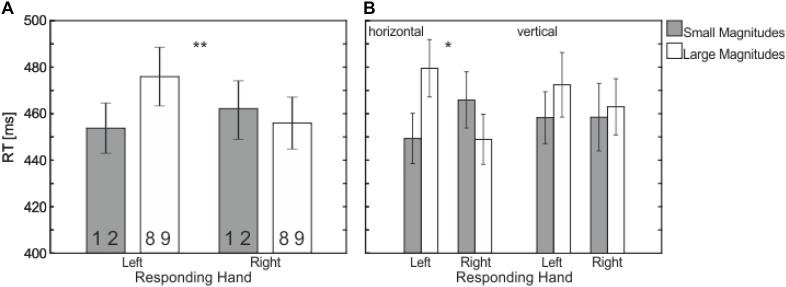
Mean RT (in ms) showing the significant interaction between **(A)** Responding Hand (left vs. right) and Numerical Magnitude (small vs. large); **(B)** Responding Hand (left vs. right) and Numerical Magnitude (small vs. large) depending on Response Dimension (horizontal vs. vertical). There is a SNARC effect only occurring in the horizontal response dimension (**B**, left side) and not in the vertical one (**B**, right). Statistically significant differences are marked by asterisks (^∗∗^*p* < 0.01, ^∗^*p* < 0.05).

In addition, there was an interaction between *Responding Hand* and *Numerical Magnitude*, *F*(1,21) = 12.21, *p* = 0.002, $\eta_{p}^{2}$ = 0.368, – the SNARC effect (see **Figures 1**, **3**) – with faster left-hand responses to smaller magnitudes (453 ms) compared to larger magnitudes (475 ms) and faster right-hand response to larger magnitudes (455 ms) compared to smaller magnitudes (462 ms).

This interaction further depends on the factor *Response Dimension* as indicated in a three-way interaction, *F*(1,21) = 4.97, *p* = 0.037, $\eta_{p}^{2}$ = 0.191. The three way interaction showed that the interaction *Responding Hand × Numerical Magnitude* is restricted to the horizontal response dimension [*F*(1,21) = 14.66, *p* = 0.001, $\eta_{p}^{2}$ = 0.411, small-left: 449 ms, large-left: 479 ms, vs. small-right: 465 ms, large-right: 448 ms]; i.e., no effect in the vertical response dimension, *F* < 1, (see **Figure 3**). None of the other main effects or interactions reached significance.

Analyses of arcsine-transformed ERs showed a main effect of *Task*, *F*(1,21) = 11.06, *p* = 0.003, $\eta_{p}^{2}$ = 0.345, indicating less errors in the magnitude judgment task (4.3%) compared to the parity judgment task (5.7%). The main effect of *Responding Hand*, *F*(1,21) = 3.95, *p* = 0.06, $\eta_{p}^{2}$ = 0.158, indicating less errors made with the left hand (4.6%), compared to the right hand (5.3%), narrowly failed to reach significance.

**FIGURE 4:**
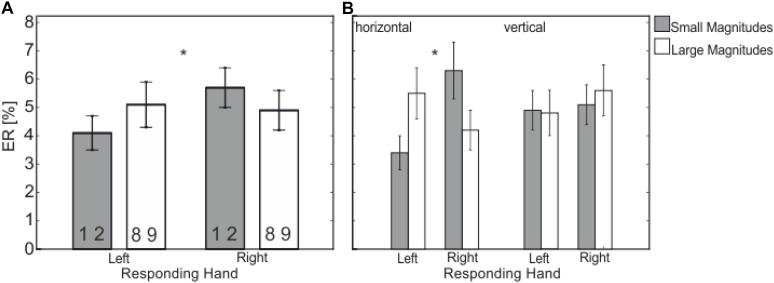
Mean ER (in %) showing the significant interaction between **(A)** Responding Hand (left vs. right) and Numerical Magnitude (small vs. large); **(B)** Responding Hand (left vs. right) and Numerical Magnitude (small vs. large) depending on Response Dimension (horizontal vs. vertical). There is a SNARC effect only occurring in the horizontal response dimension (**B**, left side) and not in the vertical one **(B**, right). Statistically significant differences are marked by asterisks (^∗∗^*p* < 0.01, ^∗^*p* < 0.05).

There was an interaction between *Responding Hand* and *Numerical Magnitude*, *F*(1,21) = 6.26, *p* = 0.021, $\eta_{p}^{2}$ = 0.230, – the SNARC effect (see **Figure 4A**) – with less errors for left-hand responses to smaller magnitudes (4.1%) compared to larger magnitudes (5.1%) and less errors for right-hand response to larger magnitudes (4.9%) compared to smaller magnitudes (5.7%).

This interaction further depends on the factor *Response Dimension* as indicated in a three-way interaction, *F*(1,21) = 5.34, *p* = 0.031, $\eta_{p}^{2}$ = 0.203. The three way interaction showed that the interaction *Responding Hand x Numerical Magnitude* is restricted to the horizontal response dimension [*F*(1,21) = 12.52, *p* = 0.002, $\eta_{p}^{2}$ = 0.374, small-left: 3.4%, large-left: 5.5%, vs. small-right: 6.3%, large-right: 4.2%]; i.e., no effect in the vertical response dimension, *F* < 1, (see **Figure 4B**).

There is a tendency that this three-way interaction also depends on the factor *Task*, *F*(1,21) = 3.59, *p* = 0.072, $\eta_{p}^{2}$ = 0.146. Further analyses showed that the *Responding Hand × Numerical Magnitude* interaction is restricted to the parity judgment task in the horizontal response dimension, *F*(1,21) = 11.45, *p* = 0.003, $\eta_{p}^{2}$ = 0.347, whereas there is no effect in the magnitude judgment task in the horizontal response dimension and in both vertical tasks, *F* < 1. None of the other main effects or interactions reached significance.

## Discussion

In magnitude and parity judgment tasks, we visually presented one-digit numbers, and varied their numerical magnitude, physical size, and responding hand. We obtained an interaction between numerical magnitude and physical size, in accordance with the *size congruity* effect, and an interaction of numerical magnitude and responding hand, in accordance with the SNARC effect. The interpretation of the responding hand × numerical magnitude interaction as a SNARC effect is further supported by the observation that this effect was restricted to the horizontal dimension. No effects corresponding to SSARC were observed. Importantly, there were no interactions involving both size congruity and SNARC effects. In order to test this null effect, BF were calculated (BF = 8.77, for the null hypothesis; Rouder, 2012). The SNARC effect, therefore, does not interact with the size congruity effect.

We also varied task conditions: RTs confirmed the parity judgment task to be harder than the magnitude judgment task (Nuerk et al., 2004; Weis et al., 2016). Noteworthy, however, is that all the other effects were independent of task. Task generality is an important characteristic of the size congruity effect (Besner and Coltheart, 1979; Henik and Tzelgov, 1982), and a defining characteristic of the SNARC effect (Dehaene et al., 1993). The task generality of our observations therefore confirms their attribution to size congruity and SNARC effects.

The size congruity effect (congruent stimuli, i.e., stimuli of which numerical magnitude and physical size are associated with the same response) were responded to faster than incongruent stimuli, of which the numerical magnitude and physical size point to different responses) replicates Santens and Verguts (2011), Experiment 2, with the same physical sizes and numerical magnitudes (1, 2, 8, and 9) different from theirs but same as in our previous studies (Weis et al., 2015, 2016,). The original set has unbalanced distances from the reference number, 5 but, as shown here, the size congruity effect does not depend on such minor imbalances.

The observation that size congruity is independent of task condition shows that the effect does not require the task-relevance of numerical magnitude, since the effect is equally strong in the parity judgment task. The result is in accordance with earlier observations on the task generality of this effect, which arises both in magnitude as well as in size judgment tasks (Besner and Coltheart, 1979; Henik and Tzelgov, 1982). These observations suggest that numerical magnitude and physical size codes are automatically evoked during the perceptual encoding of printed numbers. These codes, however, may be independently processed, and thus will not interfere until the decision stage (Santens and Verguts, 2011).

Also the SNARC effect (faster responses in SNARC compatible compared to SNARC incompatible trials, where SNARC compatibility involves compatible vs. incompatible assignment of the responding hand to the magnitude of the stimulus) is in line with previous studies (Dehaene et al., 1993; Nuerk et al., 2005; Weis et al., 2016). It is a defining characteristic of the SNARC effect that it occurs both in explicit and implicit task conditions. This observation and the restriction of our effect to the left-right orientation of the responses facilitate the interpretation of our result as a SNARC effect. The restriction to the left-right orientation is in accordance with previous studies (Ito and Hatta, 2004; Hubbard et al., 2005; Müller and Schwarz, 2007; Lourenco and Longo, 2010; Holmes and Lourenco, 2012; Hartmann et al., 2014); however, reviews by Fischer (2012) and Fischer and Brugger (2011), suggest that the SNARC effect may also appear on the vertical dimension (Ito and Hatta, 2004; Holmes and Lourenco, 2012; Hartmann et al., 2014). Some authors even reported equal strength of the SNARC effect in different orientations (Fias and Fischer, 2005; Gevers et al., 2006b; Shaki et al., 2012). A more complete experimental setup than the present one, which would vary, not only the (in)compatibility of the assignment to upper and lower buttons but also, independently, the hands with which they have to be pressed, could provide a clearer result for the vertical response condition. Such additional variation, for which complex interactions could be expected, is outside of the scope of the current article.

Crucially, no interaction involving both the size congruity and SNARC effects was obtained, which implies that the SNARC effect is independent of the conflict between numerical magnitude and physical size. This result is unexpected from the viewpoint of ideomotor theory (Hommel et al., 2001), which would predict that action effects relating to lateralized responses exacerbate the decision conflict that gives rise to the size congruity effect. The results replicate the independence for the parity task as observed by Fitousi et al. (2009) and extend it to the numerical magnitude task.

Our results, therefore, have implications for the debate of whether numbers are spatially or non-spatially represented (Santiago and Lakens, 2015). On the one hand, the size congruity effect shows that during decisions on numerical magnitude or parity, both the numerical and graphemic representation exist independently of any spatial associations. These representations interfere, suggesting they encompass magnitude/digit associations, similar to the grapheme/phoneme associations representing written letters in a reading context (Froyen et al., 2009; Lachmann et al., 2014). In this process, neither the magnitude nor the “grapheme” codes seem to be associated with a lateralized response tendency, unlike what ideomotor theory would suggest. On the other hand, the SNARC effect clearly shows associations with lateralized response tendencies. If they appear late in the process, it is likely that they are spatial in nature, as in the mental number line account of the SNARC effect (Restle, 1970; Dehaene et al., 1990) rather than conceptual, as in the polarity account (Proctor and Cho, 2006). Our conclusions, therefore, are similar to those of Fitousi et al. (2009).

We found no effect of SSARC compatibility (the compatible vs. incompatible assignment of the responding hand to the physical size of the stimulus, i.e., the interaction between Responding Hand and Physical Size). Because a size congruency effect was found, physical size must have been processed. Nevertheless, it does not lead to spatial coding of size. SSARC (in)compatibility conditions were implicit in both our tasks. This would imply that physical size does not automatically elicit a spatial coding. Fitousi et al. (2009) obtained no SSARC effect when physical size was the target of the task. We conclude that a SSARC effect for numbers is elusive. We suggest that SSARC effects may occur only when the spatial dimension is action-relevant. With disks, as in Ren et al. (2011), this may be the case, because larger disks may preferably be manipulated with the right hand.

The impossibility of independently varying size congruity, SNARC, and SSARC conditions makes the size congruity effect in principle indistinguishable from a cross-over interaction of *SNARC and SSARC compatibility* effects. Had the SSARC effect appeared in our data, the issue could be raised whether the size congruity effect should be understood as an interaction of two compatibility effects. For the current result, such deliberations are moot. Moreover, interactions between SNARC-like effects, if they occur, usually take quite a different form. In the auditory domain (Weis et al., 2016), two stimulus response incompatibilities, SNARC and SPARC (Spatial Pitch Association of Response Codes; (Rusconi et al., 2006; Lidji et al., 2007; Cho et al., 2012), showed a super-additive instead of cross-over interaction. Something similar we would have expected here, if SNARC and SSARC effects had both been obtained.

## Conclusion

In conclusion, this study confirms Fitousi et al. (2009) observations on the independence of size congruity and a SNARC effect. Thus, both effects may have different origins, the literature on the former pointing to processes in the decision stage (Santens and Verguts, 2011), and involving interactions (presumably occurring in central areas; Kaufmann et al., 2005; Ansari et al., 2006) between stimulus components that do not involve any associations with lateralized response tendencies; the latter taking place in the motor stages of processing, involving interactions between intrinsically spatially represented stimulus components to a spatial response component. These processes may take place in lateralized brain areas (Eger et al., 2003; Dormal et al., 2012; Cutini et al., 2014). Numerical representation, thus, is complex, and encompasses both spatial and non-spatial components.

## Author Contributions

All authors listed have made a substantial, direct and intellectual contribution to the work, and approved it for publication.

## Conflict of Interest Statement

The authors declare that the research was conducted in the absence of any commercial or financial relationships that could be construed as a potential conflict of interest.
